# Involvement of Atopic Dermatitis in the Development of Systemic Inflammatory Diseases

**DOI:** 10.3390/ijms232113445

**Published:** 2022-11-03

**Authors:** Misa Itamura, Yu Sawada

**Affiliations:** Department of Dermatology, University of Occupational and Environmental Health, 1-1, Iseigaoka, Yahatanishi-Ku, Kitakyushu 807-8555, Japan

**Keywords:** atopic dermatitis, Th2, immune cell, inflammation, systemic organs

## Abstract

The skin is recognized as a peripheral lymphoid organ that plays an essential defensive action against external environmental stimuli. However, continuous stimulation of these factors causes chronic inflammation at the local site and occasionally causes tissue damage. Chronic inflammation is recognized as a trigger for systemic organ inflammation. Atopic dermatitis (AD) is a chronic inflammatory skin disease that is influenced by various external environmental factors, such as dry conditions, chemical exposure, and microorganisms. The pathogenesis of AD involves various Th2 and proinflammatory cytokines. Recently updated studies have shown that atopic skin-derived cytokines influence systemic organ function and oncogenesis. In this review, we focus on AD’s influence on the development of systemic inflammatory diseases and malignancies.

## 1. Introduction

The peripheral lymphoid organ plays an essential regulation action against external environmental factors such as microorganisms, chemical exposure, and medications [1,2,3,4]. Circulating immune cells and cytokines derived from the original local inflammatory peripheral organs influence other organ functions [5,6]. Therefore, excessive immunological responses in peripheral lymphoid organs may be able to influence systemic organs.

The skin is recognized as a peripheral lymphoid tissue located in the most layered organ and is influenced by external environments [7,8]. Environmental stimuli and pathogens that are exposed to the skin cause various physiological and pathological reactions [9,10]. Chronic inflammation is a representative response to these environmental stimuli, and repeated exposure to external pathogens [11,12]. The chronic inflammatory reaction exceeds local site inflammation and occasionally develops into systemic inflammatory diseases, such as cardiovascular diseases [13,14]. Because the skin is placed in the outermost and is often exposed to the external environment or microorganisms, chronic skin inflammation may become a source of circulating inflammatory cytokines and activated immune cells, which are responsible for systemic inflammatory responses. Moreover, recently updated studies have shown that chronic inflammatory skin diseases become the trigger for the development of systemic inflammatory diseases [15].

Atopic dermatitis (AD) is an inflammatory skin disease, and recent studies have elucidated the importance of local atopic skin inflammation as a trigger for inflammation of various systemic organs and oncogenesis by the Th2-mediated immunological microenvironment. This review discussed the importance of AD for systemic inflammatory diseases and the possible pathogenic role of AD.

## 2. Pathogenesis of AD

**Figure 1:**
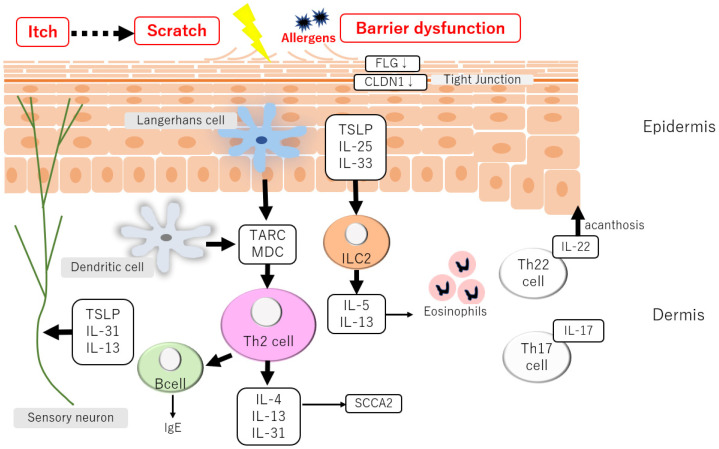
Pathogenesis of atopic dermatitis. Skin barrier dysfunction is a trigger for skin inflammation in AD. The following Th2 chemokines and cytokines production establishes the chronic inflammation in the skin recognized as the characteristics of clinical manifestation of AD.

Intercellular lipids in the stratum corneum maintain the skin barrier, and they comprise the main components, such as ceramide [16] (Figure 1). Patients with AD have decreased ceramide content in the stratum corneum and decreased filaggrin or function loss due to gene mutations [17,18]. The epidermis also functions as an important barrier to external environmental factors. Tight junctions are representative components of the adhesive structure between epidermal cells, which restricts the invasion of the outside substrates into the skin [19,20]. A decrease in the constituents of tight junctions has been identified in AD [21].

In AD, skin barrier failure is a significant contributor to chronic skin inflammation [17,18]. Type 2 immune response-mediated inflammation is caused by the synthesis of thymic stromal lymphopoietin (TSLP), interleukin (IL)-25, and IL-33 by epidermal keratinocytes [22,23,24,25]. Th2 cells migrate to skin lesions in part due to the synthesis of the thymus and activation-related chemokines and macrophage-derived chemokines in atopic skin lesions [26,27]. Additionally, in AD, IL-22 helps develop epidermal thickness [28,29,30]. IL-31 plays an essential role in the development of itch in AD [31] and neutralizing antibody treatment impairs the symptoms of AD [32,33]. Each ethnicity has a specific immune cell composition for AD. In particular, Asians have a higher Th17 cell frequency, and in an animal model of AD, Th17 cells play a vital role in the worsening of dermatitis.

## 3. AD and Systemic Inflammatory Diseases

AD is a prolonged chronic skin inflammation, which is easily speculated for the development of systemic organ inflammation and cancers. This section introduces representative systemic inflammatory disorders related to atopic skin inflammation, such as cerebrovascular diseases, endometriosis, hypothyroidism, renal dysfunction, liver dysfunction, osteoporosis, cancers, inflammatory bowel diseases, dementia, type I diabetes, and depression.

## 4. Cerebrocardiovascular Diseases

Cerebrocardiovascular diseases are the most important issue for clinicians in the management of skin inflammation, and the importance of these risks has been investigated in various countries. Several epidemiological studies have identified the influence of the presence of AD and the severity of atopic skin inflammation on the risk of cardiovascular disease.

Two clinical studies have been conducted in Asian populations. A total of 301 AD patients in the Taiwanese population were investigated, and a multivariate adjustment examination revealed that AD patients aged 20 or older were at a higher incidence of ischemic stroke [13]. The severity of AD is related to a higher risk of ischemic stroke. The hazard ratios for increased ischemic stroke risk in the severity of AD with mild, moderate, and severe had 1.20 (no significant difference), 1.64, and 1.71, respectively [13].

A Korean population study also demonstrated that AD patients in all aged populations had higher risks of myocardial infarction (hazard ratio (HR) = 9.43), angina (HR = 5.99), and stroke (HR = 10.61) [14].

In the European population, four studies have reported evidence of AD being related to the risk of cardiovascular diseases. A Danish population of 5,762,813 AD patients aged 15 years or older were identified, and those with severe AD receiving systemic treatment had higher risks of ischemic stroke (incidence rate ratio [IRR]: 1.51) and cardiovascular mortality (IRR: 1.46) [34], while mild AD showed a decreased risk of cardiovascular events, suggesting that lifestyle factors in severe AD seemed to be increased risk of cardiovascular disease [34].

In total, 387,439 AD patients aged 18 years or older were identified. Severe atopic eczema demonstrated a 20% higher risk of stroke (HR = 1.22, 95% confidence interval [CI] = 1.01–1.48) and a 70% increased risk of heart failure (HR = 1.69, 95% CI = 1.38–2.06) [35]. Several AD patients also had a higher cardiovascular event risk [35].

A study of 104,832 AD cases in a Swedish population aged 15 years or older has revealed that AD was related to angina pectoris risk. The odds ratio (OR) was 1.13 and the 95% CI was between 1.08 and 1.19 [36]. Severe AD was related to the risk of ischemic stroke (OR = 1.19, 95% CI = 1.07–1.33) [36].

Additionally, a study of 8686 patients with AD in a Danish population aged 18 years or older has demonstrated that AD was related to a higher fatal risk of cardiovascular events (HR = 1.45, 95% CI = 1.07–1.96) [37].

A systematic review has indicated that AD was associated with angina risk (risk ratio [RR] = 1.18, 95% CI = 1.13–1.24), myocardial infarction (RR = 1.12, 95% CI = 1.00–1.25), and stroke (RR = 1.10, 95% CI = 1.03–1.17) [38].

Essential filaggrin gene mutations, which were observed in AD aged 20 years or older, were related to the ischemic stroke risk (OR = 1.15, 95% CI = 1.02–1.30) [39]. Since the detailed molecular mechanisms of direct filaggrin-mediated ischemic stroke event have not been elucidated, this might simply indicate that filaggrin-mediated skin barrier dysfunction enhanced AD skin inflammation, leading to the development of vascular events.

Vascular inflammation is related to an increased Th2 immune response. AD patients increased the risk of cardiovascular disease [40]. Importantly, dupilumab treatment negatively regulates atherosclerosis-related genes in AD, suggesting the possible importance of systemic treatment for AD in the regulation of positive drivers of vascular inflammation [40].

Furthermore, IL-1 cytokines are released by external environmental stimuli and are recognized as worsening factors of atopic skin inflammation. IL-1 cytokines were upregulated in AD skin. IL-1β enhances TSLP production mediated by the NF-κB signaling pathway, in addition to reducing filaggrin expression in the epidermis [41]. Based on the importance of IL-1 cytokines in AD’s pathogenesis, IL-1 cytokines are also key factors in the pathogenesis of cardiovascular diseases. NLRP3 is highly expressed in atherosclerotic plaques, and cholesterol crystals were potential inducers for IL-1β release from atherosclerotic plaques [42]. The IL-1β blockade of canakinumab, which is not an application for AD, reduced vascular inflammation in patients with cardiovascular disease [43] and reduced recurrent cardiovascular events [44].

## 5. Endometriosis

Endometriosis is a benign disease in which the endometrium develops outside the uterus in an estrogen-dependent manner, while it also has malignant tumor-like properties exhibiting the characteristics of metastasis. Endometrial epithelial cells and stromal cells infiltrate normal muscles and connective tissues with endometrial cell proliferation and detachment in line with the female hormone cycle during menstruation, leading to tissue fibrosis, organ adhesions, and induration.

In a meta-analysis of 1821 AD patients in females between the ages of 15 and 45 years, AD was observed to be related to a higher risk of endometriosis (OR = 1.32, 95% CI = 1.09–1.55) [45].

Endometriosis develops partly because of IL-4 expression. Immunostaining of endometriotic tissues revealed IL-4-positive cells in the stroma [46]. Although the precise molecular processes of Th2 remain unknown, IL-4 in peritoneal fluid is elevated in endometriosis patients [47]. The IL-4-mediated proliferative effect was canceled by the anti-IL-4 receptor treatment, suggesting that IL-4 may have a positive regulatory role in endometriosis development. IL-4 enhances endometriotic stromal cell proliferation [46]. Endometriosis is also affected by IL-23, and IL-23 was increased in the peritoneal fluid of endometriosis patients [48].

IL-33 also contributes to endometriosis development [49]. IL-33 levels increased the inflammatory response of endometriosis and developed the proliferation of endometrial cells mediated by group 2 innate lymphoid cells [49]. Consistently, IL-33-neutralizing antibody treatment impairs the inflammatory response in endometriosis [49].

## 6. Hypothyroidism

The importance of hypothyroidism risk in AD patients has been elucidated; however, the number of clinical studies is limited. A United Kingdom-based population analysis of 173,709 patients with AD in an all-ages population demonstrated that AD significantly increased the risk of developing hypothyroidism (HR = 1.17, 95% CI = 1.09–1.25) [50].

IL-4 was upregulated in peripheral blood mononuclear cells in Grave’s disease [51]. Overexpressing IL-4 in thyrocytes in mice worsens hypothyroidism under a restricted iodine diet due to increased inflammatory cell infiltration in the thyroid [52], suggesting that certain conditions, such as a low dietary intake of iodine in patients with AD, might cause the development of hypothyroidism.

## 7. Renal Dysfunction

A clinical study has investigated chronic kidney disease risk in AD. A United Kingdom-based population analysis has reported that 56,602 chronic kidney disease patients had a history of atopic eczema aged 25 years or older (OR = 1·14, 95% CI = 1.11–1.17) [53].

IL-4 inhibition impairs renal fibrosis [54]. A mouse model of unilateral ureteral ligation-induced chronic kidney disease has demonstrated that the frequency of IL-4-producing cells was increased in the peripheral blood, and Il4-deficient mice exhibited impaired renal fibrosis mediated by the Myd88 signaling pathway [55]. Furthermore, IL-33 plays a role in chronic kidney disease development. Serum IL-33 levels were increased in chronic kidney disease patients and exhibited unfavorable survival [56]. Hence, the Th2 immune profile contributes to chronic kidney disease pathogenesis.

## 8. Liver Dysfunction

The risk of fatty liver disease in AD patients under 1 year of age was examined in a Japanese population-based study. Of the individuals with AD in Japan, 17.6% had non-obesity-related fatty livers [57].

Th2 cytokines are involved in fatty liver disease development. IFN-γ deficiency in mice advances the development of nonalcoholic steatohepatitis (NASH) in a transforming growth factor-β (TGF-β) and IL-13 signaling-dependent manner [58]. Consistently, TGF-β and IL-13 inhibition impairs non-alcoholic fatty liver disease-associated fibrosis [58]. By contrast, IL-4 itself may not be potent as a positive driver for fatty liver disease. Therefore, IL-13-involved skin inflammation, such as AD, may trigger the development of the fatty liver.

## 9. Osteoporosis

The association between the osteoporosis risk and AD has long been discussed. The incidence of osteoporosis increases in patients with AD. According to a population-based study involving 35,229 AD patients aged 20–49 years, osteoporosis was more common in AD patients (1.82 per 1000 person years) than that in non-AD patients (0.24 per 1000 person years) [59].

Additionally, a Korean population analysis of 311 patients with AD aged from 19 to  50 years has demonstrated that bone mineral density at the lumbar spine decreased in male patients with AD [60].

Although several studies have indicated that the possible osteoporosis risk in AD is associated with glucocorticoid treatment [61], recent studies have demonstrated the important incidence of osteoporosis in AD itself. Altogether, 526,808 AD patients aged 18 years or older were investigated to demonstrate an increased risk of the hip (HR = 1.10), pelvic (HR = 1.10), spinal (HR = 1.18), and wrist (HR = 1.07) fractures. The severity of AD was related to the fracture risk in spinal (HR = 2.09), pelvic (HR = 1.66), and hip (HR = 1.50) fractures, excluding the influence of oral glucocorticoid treatment [62].

Systemic treatment of AD impaired the risk of fracture and indicated that While disease-modifying antirheumatic medications (HR = 0.71; 95% CI = 0.53–0.90) and phototherapy (HR = 0.73; 95% CI = 0.56–0.95) treatment decreased the risk of fractures, severe AD was linked to a higher risk of fractures (HR = 1.31, 95% CI = 1.08–1.59) [63].

The mechanism of Th2 cytokine production in osteoporosis remains unclear. The overexpression of IL-4 reduced bone formation by osteoblasts and caused osteoporosis of both cortical and trabecular bones [64]; although, IL-4 inhibited the receptor activator of nuclear factor kappa-Β ligand (RANKL)-induced osteoclastogenesis mediated by c-Fos and NFATc1 expression in osteoclasts [65]. The non-RANKL-mediated osteoporosis mechanism might cause the pathogenesis of osteoporosis in patients with AD.

## 10. Cancers

Because cancer accelerates the development of tumor cells under Th2 immunological conditions [66], atopic skin inflammation may be a disadvantage in the development of malignant tumors. Moreover, several clinical studies have reported an increased risk of cancer in patients with AD.

Altogether, 15,666 patients with AD in Sweden have identified an increased future risk of cancer by 13% [67]. Representative malignancy risks were cancers of the brain (standardized incidence ratio [SIR] = 1.6, 95% CI = 1.1–2.4), lungs (SIR = 2.0, 95% CI = 1.3–2.8), esophagus (SIR = 3.5; 95% CI = 1.3–7.7), pancreas (SIR = 1.9, 95% CI = 1.0–3.4), and lymphoma (SIR = 2.0, 95% CI = 1.4–2.9) [67].

A United Kingdom-based study of 4,518,131 patients with AD has reported that the increased IRRs (excluding non-melanoma skin cancers) were 1.49 for cancer (95% CI = 1.39–1.61), 1.46 for non-melanoma skin cancers (95% CI = 1.27–1.69), 1.74 for melanoma (95% CI = 1.25–2.41), and 2.21 for lymphoma (95% CI = 1.65–2.98) [68].

A study of 31,330 patients with AD in the Danish population has demonstrated an increased risk of basal cell carcinoma (BCC) (SIR = 1.41, 95% CI = 1.07–1.83) and squamous cell carcinoma (SCC) (SIR = 2.48, 95% CI = 1.00–5.11) in patients with AD [69]. Meanwhile, a study of 557 patients with AD aged 18 years or older in the Netherlands has reported that the future risk of SCC was 13.1 (95% CI = 6.5–19.7) [70], and a meta-analysis has revealed a significantly increased risk of BCC as well (SRR = 1.34, 95% CI = 1.03–1.75) [71].

Th2 immunity is essential for oncogenesis and plays an important role in the downregulation of antitumor immune responses [72]. CCL17 overexpression indicated tumor development and lung metastasis in a melanoma model with subcutaneous or intravenous melanoma cell injection [72], leading to an increased risk of malignant tumor development in the Th2-dominant immune environment [72].

## 11. Inflammatory Bowel Diseases

AD increases the risk of inflammatory bowel diseases. A United Kingdom-based study with a population of 10,788 patients with AD has identified an association between AD and the risk of inflammatory bowel disease (OR = 1.107, 95% CI = 1.035–1.183) [73]. In particular, AD was linked to an increased risk of ulcerative colitis (OR = 1.149; 95% CI = 1.018–1297) [73]. Additionally, a German cohort analysis has revealed an elevated risk of inflammatory bowel illnesses among patients with AD aged 40 years or younger (*n* = 49,847). Ulcerative colitis had a higher RR of 1.25 (95% CI = 1.03–153) and Crohn’s disease had a RR of 1.34 (95% CI = 1.11–1.61) [74]. This link is also supported by findings from a meta-analysis of 95,291,110 patients with AD in a meta-analysis (OR = 1.35, 95% CI = 1.05–173) [75].

Although the detailed mechanism remains unclear, IL-4-deficiency impaired dextran sulfate sodium-induced colitis [76]. Since IgG2- and IgG3-producing cells were increased in IL-4-deficient mice [76], these conditions may have a beneficial effect on colitis.

## 12. Dementia

Several clinical studies have demonstrated that patients with AD have an increased risk of dementia. AD in a total of 1059 patients was identified to be associated with a future risk of dementia (HR = 2.02, 95% CI = 1.24–3.29) [50]. In particular, AD increased the risk of Alzheimer’s disease (HR = 3.74, 95% CI = 1.17–11.97) [77]. Severity was related to the risk of dementia and moderate-to-severe AD was related to the risk of dementia (HR = 4.64, 95% CI = 2.58–8.33) [77]. However, the detailed mechanisms remain unclear. Th2 cell levels are reduced in patients with AD [78]. Further investigation of the underlying mechanisms of AD mediated by atopic skin inflammation is necessary.

## 13. Type I Diabetes

The risk of type I diabetes in people with AD varies greatly depending on geographic region. The risk of AD was negatively correlated with type 1 diabetes in 760 cases in a German population under five years of age (OR = 0.71, 95% CI = 0.53–096) [79]. Atopic eczema was negatively correlated with the incidence of type 1 diabetes in 545 cases of childhood-onset diabetes under the age of 15 years in a community-based study in Norway (OR = 0.55, 95% CI = 0.35–087) [80]. A lower risk of AD among 54,530 Danish twins with type 1 diabetes of 3–20 years age (OR = 0.23, 95% CI = 0.07–071) was identified [81]. These European populations have a decreased risk of type I diabetes in patients with AD.

By contrast, in a Taiwanese population-based study, 3386 patients with type I diabetes were observed to have a 1.40-fold greater incidence of AD (3.31 per 1000 person years) than patients without type I diabetes (2.35 per 1000 person years) [82]. Patients with type I diabetes had a greater overall risk of AD after controlling for relevant risk variables (HR = 1.76, 95% CI = 1.29–2.39) than in those without type I diabetes [82]. Although an analysis of another Asian population has not yet been conducted, different characteristics of systemic inflammatory diseases may exist in different countries.

## 14. Depression

A relationship between AD and depression has been reported. Patients with AD aged from 21 to 36 years reportedly have higher Beck Depression Inventory scores [83]. The statistical significance of AD reportedly influences psychological disorders in many countries.

In a Taiwanese population-based study, 8208 patients with AD aged 12 years or older had a higher incidence of depression (HR = 6.56, 95% CI = 3.64–1184) [84]. In a South Korean study, 1517 patients with AD had an increased risk of depression (OR = 1.79, 95% CI = 1.40–2.29) [85]. Thus, the severity of AD is related to an increased risk of depression, and moderate-to-severe AD is significantly related to depression compared with mild AD [85]. Severity was linked to depression in AD patients who had mild (HR = 1.10, 95% CI = 1.08–1.13), moderate (HR = 1.19, 95% CI = 1.15–1.23), and severe depression (HR = 1.26, 95% CI = 1.17–1.37), according to a population-based research of 526,808 individuals with AD conducted in the United Kingdom [86].

Regarding the future risk of psychological disorders in patients with AD, another Taiwanese population-based study targeting 5075 adolescents with AD aged between 10 and 17 years has also reported a higher incidence of bipolar disorder (HR = 2.51, 95% CI = 1.71–3.67) and major depression (HR = 2.45, 95% CI = 1.93–3.11) [87]. A South Korean study involving 72,435 adolescents in middle and high school has identified relationships between AD and suicidal ideation (OR = 1.26, 95% CI = 1.16–1.36) and suicide attempts (OR = 1.29, 95% CI = 1.13–1.49) [88].

Additionally, a study of 8602 Danish children with AD aged 10 years prior to the diagnosis date showed an association with a history of maternal depression (OR = 1.18, 95% CI = 1.12–1.26) [89].

Antidepressant (HR = 1.19, 95% CI = 1.04–1.36) and anxiolytic use (HR = 1.72, 95% CI = 1.57–1.90) [90] was also reportedly associated with hospital-diagnosed AD in Danish children with AD (n = 14,283).

Additionally, a United Kingdom population-based study of 11,181 children with AD has reported that severe AD was related to depression (OR = 2.38, 95% CI = 1.21–4.72) [91]. A systematic review has also revealed that AD in children and adults increases the risk of depression (OR = 2.19; 95% CI = 1.87–2.57) [92].

Although the detailed molecular mechanism of AD and psychological disorders remains unclear, higher IL-4 and IL-13 serum levels (Th2) were also observed in a group with a major depressive disorder [93]. Dupilumab treatment impairs anxiety and depressive symptoms [94,95], suggesting the possible importance of systemic therapy against the psychological disorder development in addition to a possible role of Th2 immune response in the pathogenesis of psychological disorders.

## 15. Summary of Inflammatory Diseases in AD

Atopic skin inflammation influences systemic organ dysfunction. Since a limited number of studies have been conducted to elucidate molecular-based mechanisms of AD-mediated inflammatory responses to inflamed systemic organ dysfunction, further investigations are desired to be clarified the detailed molecular mechanisms of AD-associated systemic organ failure.

Because the skin is a large surface covering the human body, atopic skin inflammation is easily speculated to extend into systemic organ inflammation. Several inflammatory diseases are impaired by systemic therapy against AD and reduced risks. These findings suggest that atopic-mediated systemic organ inflammation negatively regulates the development of systemic inflammatory responses and organ dysfunction. Therefore, the key factors in the pathogenesis of AD and systemic inflammatory disorders highlight the importance of systemic treatment against atopic skin inflammation.

Interestingly, different risk outcomes of AD depending on the country were observed. Type I diabetes may be a representative disease, and we might need to consider the risk of systemic inflammatory diseases based on the characteristics of AD, whose immunological pathogenesis differs worldwide.

## Data Availability

Not applicable.
